# Complete genome for *Actinobacillus pleuropneumoniae* serovar 8 reference strain 405: comparative analysis with draft genomes for different laboratory stock cultures indicates little genetic variation

**DOI:** 10.1099/mgen.0.000687

**Published:** 2021-11-24

**Authors:** Janine T. Bossé, Yanwen Li, Liza Miriam Cohen, Marc Stegger, Øystein Angen, Sonia Lacouture, Marcelo Gottschalk, Liancheng Lei, Miriam Koene, Peter Kuhnert, Aloka B. Bandara, Thomas J. Inzana, Matthew T. G. Holden, David Harris, Olusegun Oshota, Duncan J. Maskell, Alexander W. Tucker, Brendan W. Wren, Andrew N. Rycroft, Paul R. Langford, on behalf of the BRaDP1T consortium

**Affiliations:** ^1^​ Section of Paediatric Infectious Diseases, Department of Infectious Diseases, Imperial College London, London, UK; ^2^​ Department of Production Animal Clinical Sciences Faculty of Veterinary Medicine, Norwegian University of Life Sciences, Ås, Norway; ^3^​ Department of Bacteria, Parasites and Fungi, Statens Serum Institut, Copenhagen, Denmark; ^4^​ Groupe de Recherche sur les Maladies Infectieuses du Porc, Faculté de Médecine Vétérinaire, Université de Montréal, Québec, Canada; ^5^​ College of Veterinary Medicine, Jilin University, Changchun, P.R China; ^6^​ Wageningen Bioveterinary Research, Lelystad, The Netherlands; ^7^​ Institute of Veterinary Bacteriology, Vetsuisse Faculty, Universität Bern, Bern, Switzerland; ^8^​ Virginia-Maryland Regional College of Veterinary Medicine, Blacksburg, USA; ^9^​ The Wellcome Trust Sanger Institute, Cambridge, UK; ^10^​ Department of Veterinary Medicine, University of Cambridge, Cambridge, UK; ^11^​ Faculty of Infectious & Tropical Diseases, London School of Hygiene & Tropical Medicine, London, UK; ^12^​ Department of Pathology and Pathogen Biology, The Royal Veterinary College, Hatfield, UK; ^‡^​ Present address: College of Veterinary Medicine, Long Island University, Brookville, USA; ^§^​ Present address: School of Medicine, University of St Andrews, St Andrews, UK

**Keywords:** *Actinobacillus pleuropneumoniae*, serovar 8 reference genome, SNPs

## Abstract

We report here the complete genome sequence of the widely studied *Actinobacillus pleuropneumoniae* serovar 8 reference strain 405, generated using the Pacific Biosciences (PacBio) RS II platform. Furthermore, we compared draft sequences generated by Illumina sequencing of six stocks of this strain, including the same original stock used to generate the PacBio sequence, held in different countries and found little genetic variation, with only three SNPs identified, all within the *degS* gene. However, sequences of two small plasmids, pARD3079 and p405tetH, detected by Illumina sequencing of the draft genomes were not identified in the PacBio sequence of the reference strain.

## Data Summary

Raw sequence data were submitted to the European Nucleotide Archive (http://www.ebi.ac.uk/ena) under study accession numbers PRJEB2343 and PRJEB10244. Individual accession numbers for each sequence are reported in Table 1. The complete closed genome of strain 405, generated by PacBio sequencing, has been deposited in GenBank with accession number CP078508. The complete sequence of the p405tetH plasmid has been deposited in GenBank under accession number MZ436971.

**Table 1.** Information on sources of *A. pleuropneumoniae* strain 405 used for genome sequencing in this study

Strain ID	Country	University/Institute^a^	Source/year^b^	Culture^c^	Extraction^d^	Accession number^e^
405D	Denmark	NVI/DTU	Original Stock/1984	BHI	FastPrep	ERS155401
405C	Canada	Montréal	Minnesota/2011	PPLO-Y-S	P-C-I	ERS155397
405Ch	China	Jilin	CIVDC/2004	BHI	AxyPrep	ERS155399
405H	Netherlands	WBVR	NVI/Unknown	Chocolate	Qiagen	ERS155398
405S	Switzerland	Bern	UK /2004; NVI/DTU/2004	BHI	FastPrep	ERS155400
405U	USA	VMRCVM	Montréal (Mittal)/2003	TSB-Y	Qiagen	ERS155396

a) Laboratory that supplied the isolate/gDNA for sequencing. NVI/DTU = Ø. Angen, when at the National Veterinary Institute, Technical University of Denmark; Montreal=M. Gottschalk, Université de Montréal; Jilin=L. Lei, Jilin University; WBVR=M. Koene, Wageningen Bioveterinary Research - formerly the Central Veterinary Institute (CVI), part of Wageningen University and Research; Bern=P. Kuhnert, Universität Bern; VMRCVM=T. Inzana, Virginia-Maryland Regional College of Veterinary Medicine. Note that 405D and 405S were supplied as cultures (lyophilized or on transport swab, respectively) for preparation of DNA at Imperial College London, whereas purified genomic DNA was provided for the remaining stocks.

b) Source from which each laboratory obtained the reference strain and the year it was obtained. Minnesota=Veterinary Diagnostic Laboratory, University of Minnesota; CIVDC=China Institute of Veterinary Drug Control, Beijing; Original Stock=isolated in Ireland, provided to R. Nielsen (NVI) by P.J. O’Connor [13]; Unknown=part of a culture collection held by E.M. Kamp at the CVI, Lelystad; obtained from R. Nielsen (NVI) prior to 1987 (Kamp *et al*., 1987); UK=P. Langford, Imperial College London, this stock was obtained the same year (i.e. 2004) from Ø. Angen at the NVI; Montréal (Mittal)=K. Mittal, Université de Montréal. It should be noted that prior to 2011, the stock held at the Université de Montréal in the laboratory of M. Gottschalk had been the same as that of K. Mittal, but sometime between 2003 and 2011 a problem with this stock became apparent (not producing expected serovar-specific amplicon using the Zhou *et al*., 2008 mPCR) and a replacement stock was acquired from the University of Minnesota.

c) Culture medium used for growth of strain 405. PPLO-Y-S=PPLO agar (Difco) supplemented with 10 % yeast extract and 5 % horse serum; BHI=Brain Heart Infusion (Difco); Chocolate=Columbia chocolate agar (WBVR, cat. BM248)); TSB-Y=Tryptic Soy Broth (Difco) containing 0.6 % yeast extract (Difco). All media were supplemented with nicotinamide adenine dinucleotide (5–10 µg ml^−1^).

d) Extraction method used to prepare genomic DNA for sequencing. P-C-I=phenol-chloroform-isoamyl; AxyPrep Bacterial Genomic DNA Miniprep kit (Axygen); FastPrep=FastDNA spin kit (MP Biomedicals); Qiagen=DNeasy Blood and Tissue kit (Qiagen).

e) Accession numbers provided are for the raw read files of the draft genomes used for SNP analysis, which were deposited in the European Nucleotide Archive. The complete annotated genome for strain 405 (generated using the 405D sample) is available in GenBank under the accession number CP078508, with raw sequence reads available in ENA under accession number ERS809856.

**Impact Statement**

*
Actinobacillus pleuropneumoniae* is a bacterium that causes a lung disease in pigs responsible for substantial economic losses worldwide. The bacterium is surrounded by one of 19 sugar capsules, and this determines the serovar. Factors associated with severity of disease are not uniformly present in all serovars, and identifying serovars helps determine which vaccines should be used in a region/country. To help researchers accurately identify serovars, and to characterise important differences between them, ‘reference’ strains of each of the 19 serovars are available. However, mistakes in strain identification can lead to results being ascribed to the wrong strain/serovar, and/or genetic changes can accumulate during culture and storage that can affect results. In the case of *A. pleuropneumoniae *, it has been reported that two different reference strains of serovar 8 with different characteristics have been circulating in worldwide collections. In this study, we have compared the genome sequences of reference serovar 8 strains from six countries from three continents. Our results confirm that stocks held by the six laboratories are the correct serovar 8 reference strain, with little genetic difference, and researchers can have confidence in its use in determining serovar and in other studies.

## INTRODUCTION

The porcine respiratory tract pathogen, *Actinobacillus pleuropneumoniae *, was first described as a *Haemophilu* s-like organism, isolated in 1957 from pneumonic lung lesions of pigs in Great Britain [1]. It was subsequently found to be the causative agent of severe and often fatal porcine contagious pleuropneumonia in pigs in Argentina and was initially named *Haemophilus pleuropneumoniae* [2], with Shope 4074 designated as the type strain for the species [3]. Transferred to the genus *Actinobacillus* in 1983 [4], *A. pleuropneumoniae* is an ecomonically important pathogen in all swine-producing countries around the world, with currently 19 recognised serovars differing in geographic distribution, though temporal shifts in dominant serovars have been reported in some countries [5–8]. Serological differentiation is based on antibody recognition of distinct carbohydrate antigens on the bacterial surface, mainly capsule, with cross-reactivity detected between some serovars (3/6/8/15, 4/7, or 1/9/11) expressing identical or highly related lipopolysaccharide O-antigens [9, 10]. Genetic characterisation of serovar-specific capsule genes has allowed more accurate discrimination of serovars, and molecular capsule typing is replacing serology for routine diagnostics [6, 11, 12].

Serovar 8 was first isolated in Ireland and Denmark in 1984, with the Irish strain 405 designated as the reference strain [13]. This serovar has since been found to be the most prevalent in circulation in the United Kingdom [14, 15] and Norway [16], and it has also been identified in North and South America [17, 18], Denmark [19], and Belgium. We previously reported the complete genome sequence of a genetically tractable serovar 8 clinical isolate, MIDG2331, from the UK [20]. We further showed that MIDG2331 encodes a 56 kb integrative conjugative element (ICE*Apl1*) found in a subset of serovar 8 isolates, and absent in strain 405 [21]. Draft and or complete genome sequences have been previously reported for the reference strains of many *A. pleuropneumoniae* serovars [22–24], but not strain 405.

Not all of the *A. pleuropneumoniae* serovar reference strains are available through the common culture collections, such as the American Type Culture Collection (ATCC) and National Collection of Type Cultures (NCTC), and sets of reference strains have commonly been distributed between research groups around the world. Mistakes in strain identification can lead to genotypic/phenotypic results being ascribed to the wrong strain/serovar. For example, the draft genome sequence deposited as the serovar 1 reference strain Shope 4074 (accession number NZ_AACK01000000) is actually a serovar 5 strain, as indicated by the serovar specific capsule genes present [11]. Similarly, the draft genome sequence deposited as the biovar 2 serovar 13 reference strain N273 (accession number NZ_ADOM00000000) has a capsule locus indicating it is a serovar 7 isolate and has a truncated *nadV* gene, indicating it is biovar 1 [11]. Although still the same serovar, Gram *et al*. [25] reported that a Danish field isolate of serovar 8 had been mixed up with reference strain 405 during routine lab use, leading to confusion over the reported genotype of *omlA* for this strain [19]. Furthermore, changes in reference strain genomes due to genetic drift following multiple passages in the lab have also been reported for various bacterial species, sometimes leading to phenotypic differences [26–29]. Genetic changes can also occur following extended incubation over several days in rich medium, such as when agar stabs are used for shipping bacterial strains between research groups [30].

In this study, we used PacBio sequencing technology that facilitates long DNA reads to generate a complete closed genome for the *A. pleuropneumoniae* serovar 8 reference strain, 405, using DNA from an aliquot of the original stock [13] held at the National Veterinary Institute, Technical University of Denmark. Additionally, using the Illumina platform, we generated draft genome sequences from this sample, as well as DNA from five other stocks of this strain held in different laboratories around the world in order to determine, using SNP analysis, if any genetic differences exist between them.

## METHODS

The sources of DNA for the different stocks of *A. pleuropneumoniae* strain 405 are shown in Table 1. The original stock, held in Denmark and termed 405D, was used for generation of the complete closed reference genome, using the PacBio platform, as well as for generation of a draft genome by Illumina sequencing (see below). Although DNA was prepared from cultures grown from master freezer stocks (stored at −80 °C in 15–25 % glycerol) in each laboratory, it is not known how many passages each stock may have been subjected to prior to acquisition from other groups. There were some differences in media used to grow the culture and methods to prepare the genomic DNA between the labs, as indicated (Table 1).

For draft genome sequences, paired-end libraries were generated from approximately 500 ng of genomic DNA as previously described [31, 32] and sequencing was performed at the Wellcome Sanger Institute (Hinxton, UK) on an Illumina HiSeq 2000 analyzer to obtain paired 75 bp reads. For each draft genome, the sequences underwent quality assessment using bifrost (https://github.com/ssi-dk/bifrost) and were assembled into contigs using SKESA [33].

PacBio sequence reads were assembled using HGAP v3 [34], as part of the SMRT analysis software v2.3.0 (https://github.com/PacificBiosciences/SMRT-Analysis). When picking the minimum fragment length for assembly, the fold coverage to target was set to 30 and the approximate genome size was set to 3 Mbp. Circlator v1.1.3 [35] was used to circularise the assembly, which was then polished using the PacBio RS_Resequencing protocol and Quiver v1 of the SMRT analysis software v2.3.0.

Assembled sequences were analysed for the presence of acquired antimicrobial resistance genes using ResFinder v4.1 [36], with a threshold of 90 % identity and minimum length of 60 %. The PacBio closed genome was annotated using the National Centre for Biotechnology Information’s (NCBI’s) Prokaryotic Genome Annotation Pipeline (PGAP).

Following removal of duplicated regions using NUCmer, detection of single nucleotide polymorphisms was performed using the Northern Arizona SNP (NASP) pipeline v1.2 [37] using BWA to align Illumina reads from individual draft genomes of the six stock cultures of the *A. pleuropneumoniae* serovar 8 reference strain 405 against the PacBio closed genome generated for the original stock culture (strain 405D). Positions with less than ten-fold coverage and less than 90 % unambiguous variant calls were excluded.

## RESULTS

PacBio sequencing of the 405D sample yielded 105 545 circular concensus reads, with a mean length of ~4300 bp, of which a total of 6017 reads with a mean length of ~8000 bp after error correction were used to obtain a single contig of 2323218 bp with a GC content of 41.1%, which is typical for *A. pleuropneumoniae* genomes. The genome of strain 405 is syntenic with that of the previously reported complete genome of the serovar 8 clinical strain, MIDG2331 [20]. The only differences consist of phage gene insertions, which vary between the two genomes, and the ICE*Apl1* insertion in MIDG2331 [21] which is absent from 405.

Quality control and assembly of the genomes for the six different stock 405 strains, sequenced using the Illumina platform, showed that each draft genome was of high quality, with sequencing depths of >75. For each draft genome, the reads were assembled into 41 or 42 contigs, all with a GC content of 41.0 %. Alignment of the draft genome sequences with the PacBio closed reference genome showed, after removal of duplicated regions, a core of 2208689 bp equivalent to 95.07 % of the reference chromosome across all six samples, with only three SNPs identified, all in the *degS* gene (Table 2).

**Table 2.** SNPs identified in the *degS* gene identified in genomes of different stocks of the *A. pleuropneumoniae* serovar 8 reference strain 405 and associated changes to the encoded proteins

Genome^a^	Base (137)^b^	AA (46)^c^	Base (646)	AA (216)	Base (688)	AA (230)
405C	C	Ala	A	Lys	C	Pro
405Ch	C	Ala	A	Lys	C	Pro
405D	C	Ala	A	Lys	**T**	**Ser**
405H	**A**	**Asp**	A	Lys	C	Pro
405S	C	Ala	A	Lys	C	Pro
405U	C	Ala	**G**	**Glu**	C	Pro
405D*	C	Ala	A	Gly	T	Thr

a) Identity of specific strain 405 stock used to generate draft genomes (or, in the case of 405D*, the PacBio closed genome). See Table 1 for laboratory of origin for each stock.

b) Base position in the *degS* gene relative to the start codon.

c) Amino acid at the corresponding position (shown in parentheses) in the DegS protein.

Each of the three detected nucleotide changes is associated with a nonconservative substitution in the 343 amino acid (AA) DegS protein. The sequence of the original strain 405 stock culture (both the draft 405D genome and the complete PacBio sequence) encodes a DegS protein with a serine at residue 230, whereas all of the other stock culture genomes show a proline residue at this site that appears to be conserved in all other sequenced *A. pleuropneumoniae* DegS proteins, including other serovar 8 sequences (WP_005600971, WP_039709089). Additionally, the 405H protein differs at residue 46 (an aspartic acid rather than alanine), and that of 405U differs at residue 216 (a glutamic acid rather than a lysine), from the other *A. pleuropneumoniae* DegS sequences. Whereas the *degS* genes in 405C, 405Ch and 405S encode proteins sharing 100 % identity with those from other sequenced serovar 8 isolates of *A. pleuropneumoniae* (WP_039709089).

ResFinder analysis of the assembled PacBio sequence did not identify any known resistance genes, whereas *tet*(H) (Y15510) and *sul2* (AY034138) were identified on two different small contigs in each of the draft genomes. These appeared as contigs 1 and 2 in all the SKESA assemblies, and were identified as circular contigs of 5470 bp and 4063 bp, respectively. Further analysis of the 4063 bp contig, identical in each draft genome, revealed that *sul2* is encoded on a plasmid that shows 99 % ID with pARD3079, previously described for *A. pleuropneumoniae* [38]. Analysis of the 5470 bp contig, also identical in each draft genome, revealed that the tetracycline resistance gene, *tet*(H), is encoded along with the *tetR* regulator and plasmid mobilization genes, *mobA* and *mobC*, in a novel plasmid that shares partial identity with some previously described plasmids in various *Pasteurellaceae* species, including the *A. pleuropneumoniae* plasmid p9956 [39] and pB1018 from *Pasteurella multocida* [40]. The novel plasmid in *A. pleuropneumoniae* strain 405, designated p405tetH, shares 99 % identity across the *tet*(H)/*tetR* genes, but less conservation across the mobilization genes (83 % ID), which are found in the opposite orientation in p9956 and pB1018.

## DISCUSSION

Unintentional mutations can occur in bacterial stock cultures that can affect phenotypes under study, possibly leading to contradictory results from different laboratories. When compared to the genome of the *Escherichia coli* strain MG1655 published by Blattner *et al*. [41], Freddolino *et al*. [27] found a set of seven mutations in the genome sequences of different stocks of this strain that had been acquired from the Blattner laboratory in 2003, as well as in stocks of ATCC 700926 acquired on two separate occasions; whereas an older stock (ATCC 47076) contained only a subset of the mutations compared to the published genome. For the *Campylobacter jejuni* reference strain, NCTC 11168, the sequence has been documented to vary at least 200 times over three decades, significantly affecting its phenotypic properties [29].

It has been noted that mutations can occur during transfer of bacterial strains between laboratories, when bacteria are often incubated for several days in rich medium transport stabs [30], and also during long-term preservation of stocks within culture collections [42]. Furthermore, even limited passages in the laboratory can result in mutations [26, 43], which may cause phenotypic changes.

Most *A. pleuropneumoniae* research groups have acquired their reference strain collection from other laboratories, rather than from curated culture collections such as ATCC or NCTC. Given the previous report that a Danish field isolate of *A. pleuropneumoniae* serovar 8 had been mistaken for the reference strain 405 [25], meaning it could be in circulation as the reference strain, we decided to do comparative sequence analysis of different international laboratory stocks of this strain in addition to generating a closed reference genome that will facilitate further functional genomic analysis of this serovar [44].

All of the genomes sequenced in this study had the *omlA* gene sequence previously described for strain 405 (Y12811) and not that of the Danish clinical serovar 8 isolate (U86683) mistakenly used as the reference strain in development of an *omlA*-based diagnostic PCR [19], indicating they are all true stocks of the *A. pleuropneumoniae* serovar 8 reference strain 405. Furthermore, the draft genome sequences show little genetic variation between the different stock cultures, with only three SNPs identified, all within the *degS* gene.

DegS is a serine protease which functions as a periplasmic stress sensor required for activation of the alternative sigma factor, RpoE, via degradation of the anti-sigma factor, RseA [45]. In *A. pleuropneumoniae *, both *rseA* and *rpoE* were identified as important for survival of the bacterium within the pig during acute infection [46], and RpoE was subsequently shown to be a key regulator of biofilm formation by this bacterium [47]. A *degS* deletion mutant has been generated in *A. pleuropneumoniae *, leading to increased production of outer membrane vesicles [48], but no other phenotypes were tested, and no structure/function analysis has been done for DegS in this bacterium. The DegS protein is best characterised in *E. coli *, with a crystal structure supporting domain analysis for identification of key active site residues [49]. Although the *A. pleuropneumoniae* DegS shares only 49 % identity with that of *E. coli *, regions of conservation include the alanine, lysine, and proline residues described above and found in the majority of *A. pleuropneumoniae* DegS sequences. However, none of these residues has been identified as critical for the structure or activity of the *E. coli* protein, and it is unclear if the alternate AAs would have any effect on the *A. pleuropneumoniae* DegS proteolytic activity.

Overall, the results of this study confirm that the strain 405 cultures held in stock collections in various laboratories around the world are all the correct serovar 8 reference strain. Furthermore, despite acquisition from various other laboratories over the years, with uncertain number of passages (possibly on different types of media) between transfers, only three SNPs were detected in the genomes prepared from the different cultures. That all three SNPs affect the *degS* gene, encoding a protease predicted to be involved in activation of RpoE, suggests possible selection for these mutations in response to extracytoplasmic stress(es) encountered during passage/transport/storage, rather than random genetic drift.

Some studies have indicated that PacBio-generated sequences may be less accurate than those generated with the Illumina HiSeq platform, though improvements have been achieved more recently [50, 51]. Our comparison of the Illumina- and PacBio-generated genomes for the original stock 405D culture revealed no SNPs between these sequences, indicating comparable accuracy with both platforms. It has also been previously reported that small plasmid sequences may not be identified using the PacBio platform [52, 53], and this was seen in our results, with sequences for two small plasmids (pARD3079 and p405tetH) found in all of the draft genomes, but not the PacBio sequence. It should be noted that the presence of the two plasmids will not affect the use of the strains as serovar controls, but has the potential to introduce variation into other types of studies, e.g. those involving antimicrobial resistance. The availability of the complete closed genome (and associated plasmid sequences) for the serovar 8 reference strain 405 will facilitate functional genomic analysis, as well as further comparative genome studies with other isolates of this increasingly prevalent *A. pleuropneumoniae* serovar.
